# Gel Formulation of Nabumetone and a Newly Synthesized Analog: Microemulsion as a Photoprotective Topical Delivery System

**DOI:** 10.3390/pharmaceutics12050423

**Published:** 2020-05-05

**Authors:** Fedora Grande, Gaetano Ragno, Rita Muzzalupo, Maria Antonietta Occhiuzzi, Elisabetta Mazzotta, Michele De Luca, Antonio Garofalo, Giuseppina Ioele

**Affiliations:** Department of Pharmacy, Health and Nutritional Sciences, University of Calabria, 87036 Rende (CS), Italy; fedora.grande@unical.it (F.G.); gaetano.ragno@unical.it (G.R.); mariaantonietta.occhiuzzi@unical.it (M.A.O.); mazzotta-elisabetta@libero.it (E.M.); michele.deluca@unical.it (M.D.L.); antonio.garofalo@unical.it (A.G.)

**Keywords:** anti-inflammatory drugs, nabumetone, photostability, microemulsion-in-gel, multivariate curve resolution

## Abstract

Photostability studies were performed on topical formulations containing the anti-inflammatory drug Nabumetone and an analog newly synthesized in order to achieve better photostability and pharmacokinetic profile. Stability tests, according to the International Conference on Harmonization rules, were applied on ethanol solutions and topical gel formulations of both compounds. The photodegradation profiles were monitored by Multivariate curve resolution applied to the UV spectral data. The inclusion of the compounds in microemulsion was investigated to improve light stability and, at the same time, to ensure a sustained release system for skin delivery. All the formulations in solution, gel, microemulsion, and microemulsion-in-gel were exposed to a forced irradiation of 350 W/m^2^, corresponding to a 21 kJ/m^2^ min, for up to 300 min. Photostability increased significantly for both drugs in the liquid microemulsion and microemulsion-in-gel, compared to the ethanol solution and plain gel, reaching a residual drug of 97% and 98% for Nabumetone and analog in microemulsion-in-gel, respectively. Permeation experiments on the microemulsion-in-gel showed a better performance of the analog formulated at 0.2%, compared to the same formulation of Nabumetone at 0.7%. These results highlight the potential of the designed matrices as delayed drug delivery systems along with the use of lower drug doses leading to reduced side effects.

## 1. Introduction

Nabumetone (4-(6-methoxy-2-naphthyl)butan-2-one) (NA), based on a 2,6-disubstituted naphthyl-alkanone structure, is a non-acidic nonsteroidal anti-inflammatory drug (NSAID) which is rapidly metabolized in the liver to the major active metabolite 6-methoxy-2-naphthyl acetic acid (6-MNA) [1]. The side effects of this drug are well known [2,3] as well as its sensitivity to light [4]. Actually, the photo-oxidation of the side chain to 6-methoxy-2-naphthaldehyde, as a major product, and the formation of the (4-(6-methoxy-2-naphthyl)-3-buten-2-one) have been already reported [4,5]. Photosensitivity represents an important limit throughout the various stages of production, storage and distribution as well as during clinical application, particularly in local skin formulations [6]. It seems necessary to develop new strategies aimed at preventing or minimizing photodegradation of NA, also considering that the use of protective containers or packaging has proved to be insufficient to guarantee light stability [7,8,9].

**Figure 1:**
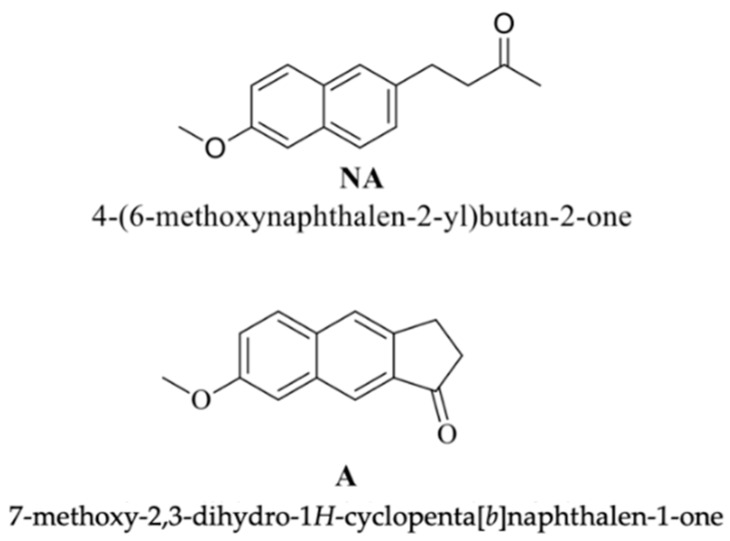
Chemical structures of Nabumetone (4-(6-methoxy-2-naphthyl)butan-2-one) (NA) and its analog A.

For this aim, structural modifications on NA were attempted to improve drug stability and pharmacokinetics and reduce side effects. Accordingly, a new derivative A was designed in which the butanone chain was replaced by a condensed cyclic ketone moiety (Figure 1). This analog was designed considering the characteristics of the receptor binding-site, with the aid of computer-assisted approaches, thanks to the availability of several data on the crystal structure of the cyclo-oxygenase-2 protein [10]. Furthermore, a calculated log P of 2.4 for compound A reflects a hydrophobicity adequate to allow good absorption and permeation after local administration [11].

Furthermore, when the pharmacokinetics of a drug is not favorable and at the same time the absorption through the skin is slow, a system to protect the drugs from prolonged sun exposure after application should be designed. Some examples are proposed in the studies of incorporation into supramolecular systems, such as cyclodextrins, liposomes, and niosomes, often supported by the addition of antioxidants and solar filters [12,13,14,15,16,17]. Recent investigations suggest the use of microemulsions (ME) as dispersion systems to realize photoprotective carriers for drugs [18,19,20]. These systems are solutions optically isotropic and thermodynamically stable with droplet size in the submicron range. Usually, they consist of oil phase and aqueous phase with the addition of surfactants and cosurfactants. Some advantages offered by ME include high drug solubilization capacity, enhancement of skin permeation for both hydrophobic and hydrophilic drugs, easy manufacturing, and a prolonged shelf life [21,22]. In 2018, the entrapping of NA in ME has been proposed by Jagdale et al. [23] to optimize transdermal micro-emulgel delivery of the drug for the treatment of arthritis.

In this work, the light stability of NA and its analog A were investigated in solution and gel formulations, according to the international International Conference on Harmonization (ICH) rules [24]. Photoprotection studies were performed by entrapping the compounds into liquid ME, solution and gel formulation (MEG). The photodegradation profiles of the species in the photodegradation experiments were monitored by UV spectrophotometry and the spectral data were processed by Multivariate Curve Resolution (MCR). This chemometric procedure was chosen because particularly suitable to follow the kinetic processes of a chemical transformation, allowing to estimate spectra and concentration profiles of the components involved [25,26,27,28,29,30]. The delivering behavior of the novel gel formulations was investigated by ex-vivo permeation studies using rabbit ear skin and vertical Franz diffusion cells. The transdermal administration represents an attractive and accessible route for testing drug absorption overcoming problems associated with oral or parenteral administration, such as a low bioavailability due to first-pass metabolism.

Topical NSAIDs represent the main clinical device to treat the inflammatory status and are often used in the therapy of rheumatic and non-rheumatic diseases of the muscle-skeletal system, such as accidents during sport activities. The most important advantage of topical NSAIDs is the lack of serious adverse effects associated with systemic NSAIDs, particularly in elderly patients. Oral treatment with NSAIDs has been in fact associated with increased gastrointestinal (GI), renal, and cardiovascular toxicity [31]. Evidence indicates that topical formulations can achieve therapeutic drug concentrations in localized tissues by keeping serum drug levels low and potentially avoiding systemic toxicity [32]. In this context, the development of photostable formulations for topical use of newly synthesized NSAIDs could lead to the availability of drugs with effective biological activity, high stability, and reduced side effects.

## 2. Materials and Methods

### 2.1. Chemicals, Instruments and Software

NA, propylene glycol, microcrystalline cellulose, Brij^®^ 97, and isopropyl myristate were purchased from Sigma-Aldrich (Milan, Italy). Ethanol and methanol were from J.T. Baker (Deventer, Holland).

UV spectra were recorded by using a Perkin-Elmer Lambda 40P Spectrophotometer by setting the following instrumental conditions: λ range 200–450 nm, scan rate 1 nm/s; time response 1 s; spectral band 1 nm. Spectral acquisition and elaboration were made by using the dedicate software UV WinLab^®^ (Perkin-Elmer, Waltham, MA, USA). A light cabinet Suntest CPS+ (Heraeus, Milan, Italy) equipped with a Xenon lamp was used to perform the photodegradation experiments. The ID65 standard filter was set to simulate sunlight in a spectral range between 300 and 800 nm. Multivariate analysis was performed by the software Matlab^®^ computer environment (Mathwork Inc., version 7, Torino, Italy).

### 2.2. Chemistry

Compound A was prepared in good yield and high purity by polyphosphoric acid catalyzed Friedel-Crafts intramolecular cyclization of 3-(6-methoxynaphthalen-2-yl)propanoic acid [33]. Chemical and analytical data are reported in “Supplementary Materials”.

In detail, a mixture of 3-(6-methoxynaphthalen-2-yl)propanoic acid (100 mg, 0.43 mmol) and polyphosphoric acid (1 g) was heated to 110 °C for 1 h. After cooling, the mixture was poured into cold water and stirred for an additional 30 min before extraction with ethyl acetate. The organic layer was successively washed with NaHCO_3_ saturated solution, water and brine then dried over magnesium sulfate. The evaporation of the solvent left a residue which was purified by column chromatography on silica gel (*n*-hexane/ethyl acetate 6:1, as eluent) to give 73 mg (79% yield) of pure compound A.

### 2.3. Sample Preparation

A calibration set, including ten standard one-component solutions of NA and A in ethanol in a concentration range of 0.5–10.0 μg/mL, was prepared and used to establish the mathematical relationships between concentration and respective analytical signals. Thus, different MCR procedures were applied to the spectrophotometric data of these solutions. A set of five ethanol solutions (prediction set) containing NA or A in the same concentration range used for the calibration set, was prepared in order to apply the analytical methods defined and used to calculate statistical effectiveness of the methods in terms of accuracy and precision. A last set of diluted solutions was prepared to measure the Limit of Detection (LOD) and the Limit of Quantitation (LOQ) of the chemometric procedures in calculating the concentration of NA and A in all the samples.

Photodegradation test was performed on standard solutions of NA and A, prepared in ethanol at the concentration of 5.0 μg/mL, in consideration of the low solubility of the compounds in water.

According to the European Pharmacopoeia procedures [34], gel formulation (20 g) of both compounds were prepared by emulsifying the drug 0.20 g (1% *w/w*) in propylene glycol 2 g under continuous stirring for 15 min. 0.60 g of microcrystalline cellulose (gelling agent) and 17.2 g of water were then added, and the final emulsion was stirred for 50 min getting a homogeneous white gel.

ME composition was established by considering the ternary diagram phase Brij 97—isopropyl myristate—H_2_O, in absence of a cosurfactant, as reported by Wang et al. [35]. In this diagram, five single-phase regions are identified, in which two regions correspond to isotropic solution phases, one is an anisotropic lamellar liquid crystalline phase, one is an anisotropic hexagonal liquid crystalline phase and one is an isotropic micellar cubic liquid crystalline phase. In consideration of the used water content, between 65.5 and 90.5 wt. %, the prepared ME is an isotropic solution phase, i.e., an O/W microemulsion [36,37]. In the first step, the oil phase (isopropyl myristate, 0.25 g) and the Brij 97 surfactant (0.95 g) were mixed and heated at 70 °C for 3 min. The drug was solubilized in this mixture and then added to water (3.80 g) dropwise. Finally, the samples were subjected to several centrifugation cycles at 3500 rpm for 10 min, followed by 4 min of vortex mixing at 2200 rpm. The formation of ME was confirmed by the appearance of a clear and transparent isotropic solution. The final drug concentration in ME formulation was 0.7 wt. %.

In order to make ME suitable for topical application, it was incorporated into a gel matrix (MEG). The formulation was prepared by the following procedure: 4.46 g of drug-ME were added to 0.65 g of propylene glycol and 0.15 g of cellulose and stirred magnetically up to 15 min to get an opalescent homogenous gel.

### 2.4. Size and Distribution Analysis

The mean diameter of the micro-emulsion oil droplets was measured by dynamic light scattering (DLS) using a 90 Plus Particle Size Analyzer (Brookhaven Instruments Corporation, New York, NY, USA) at 25.0 ± 0.1 °C. Before measurement, micro-emulsions were appropriately diluted in distilled water and the results were directly obtained from instrument data fitting through the inverse “Laplace transformation” and the Contin methods [38]. The polydispersity index (PDI) was used as a measure of the width of size distribution. The PDI values lower than 0.3 indicated a homogenous population for colloidal systems. All the measures were done in triplicate and expressed as mean ± standard deviation.

### 2.5. Photodegradation Test

The photodegradation tests were made on all the prepared liquid and gel formulations under the following conditions: irradiation power 350 W/m^2^, corresponding to 21 kJ/m^2^ min, temperature 25 °C. The samples were analyzed just after preparation (t = 0 min) and at several exposure times (10, 30, 50, 70, 100, 130, 150, 210, 270, 300 min) by UV spectrophotometry. All laboratory experiments were carried out in a dark room to minimize additive light interferences.

Drug content in liquid samples was calculated by MCR applied on the UV spectral data recorded along the photodegradation experiments. MCR methods were previously elaborated on the NA and A ethanol solutions (calibration sets) prepared as above described. The robustness of the chemometric methods was determined by predicting the concentration of other five samples prepared in the same concentration range (prediction set). The parameter lack of fit (% lof), used to indicate the fit quality of the MCR results, was below 5% in all the experiments. LOD was calculated to be 0.0081 and 0.0093 μg/mL for NA and A, respectively. LOQ was in the range 0.078–14.3 μg/mL for NA and 0.089–13.5 μg/mL for A.

ME formulations were previously diluted with ethanol to obtain a drug concentration of 5 μg/mL at zero time. Gel formulations (0.5 g) were uniformly stratified on a glass plate to form a layer thickness of 0.25 mm and then exposed to forced irradiation. After each irradiation dose, the glass plate was sonicated in acetonitrile 25 mL for 10 min at room temperature. Then, 10 mL of the obtained suspension was centrifuged at 5000 rpm for 10 min and the supernatant was analyzed after 1:10 dilution with methanol.

### 2.6. Ex-Vivo Permeation Studies

Ex-vivo permeation studies were carried out using rabbit ear skin obtained from local slaughterhouse and vertical diffusion Franz cells for 7 h at 37 °C. This temperature value was selected according to the results reported in literature [39,40]. The receptor solutions were maintained at 37 °C in order to assure a temperature of skin surface loaded in diffusion cell in the range 32–35 °C.

The skin, previously frozen at −18 °C, was pre-equilibrated in physiological solution at room temperature for 2 h before the experiments. A circular piece of skin was placed between the receptor and donor compartment with the dermal side in contact with the receiver medium and the epidermis side in contact with the donor chamber (contact area = 0.416 cm^2^). The donor compartment was charged with an appropriate volume of sample to keep constant the drug molar concentration in particular, the amount of drug loaded in the donor compartment was 1.00 × 10^−6^ moles for the samples containing 0.2% of drug and 2.98 × 10^−6^ moles for the ones at 0.7%. The receptor compartment was filled with 5.5 mL of fresh hydroalcoholic solution (water:ethanol 1:1), maintained at 37 ± 0.5 °C and stirred by a magnetic bar. At regular time points up to 7 h, the receptor solution was removed for analysis and replaced with an equal volume of a pre-thermostated (37 ± 0.5 °C) fresh one.

The drug content in the samples was calculated by spectrophotometry. Each experiment was carried out in triplicate and the results agreed within ± standard deviation.

## 3. Results

### 3.1. Micro-Emulsion Characterization

Incorporation of the drugs into ME was set at 0.2% due to the limited solubility of A. However, a further formulation with 0.7% NA concentration was needed to reach a comparable permeation rate through the skin.

ME loaded with NA or A appeared as an isotropic and translucent solution. According with the results provided by Ingvar Danielsson and Bjorn Lindman in 1981, the prepared ME is a mixture of water, oil and surfactant resulting in an liquid isotropic system, fluid, transparent and thermodynamically stable [41]. Actually, the samples were very stable, similar size and PDI and no sedimentation, creaming or flocculation were observed for 6 months. The average oil droplet size, diameter in the range 13–23 nm, and the polydispersity index of micro-emulsions are reported in Table 1, after 1 day and six months from preparation. The loading of the drug 0.2% resulted in a decrease of mean oil droplet size, whereas the same trend was not observed in the 0.7% formulation. This behavior was probably due to the drug which at low concentrations acts as an emulsifying agent capable of reducing the oil droplet size, as described in a previous work [42]. The polydispersity index of the samples was in the range between 0.153 and 0.214, indicating a narrow oil droplet size distribution and, consequently, that the formulations had relatively homogenous dimensions.

### 3.2. Photodegradation of Liquid Formulations

**Figure 2:**
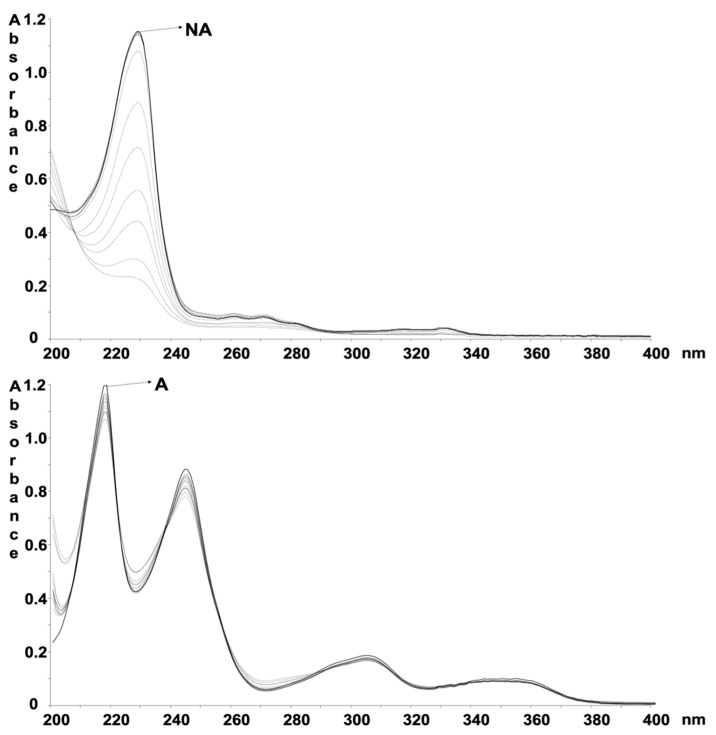
Absorbance spectra recorded on the ethanol solution of NA and A at the following exposure times: 0, 10, 30, 50, 70, 100, 130, 150, 210, 270, 300 min.

The ethanol solutions of the compounds at a concentration of 5.0 mg/mL were subjected to forced photodegradation, under the conditions above reported. The spectra, shown in Figure 2, were recorded just after the preparation and at several exposure times up to 300 min.

**Figure 3:**
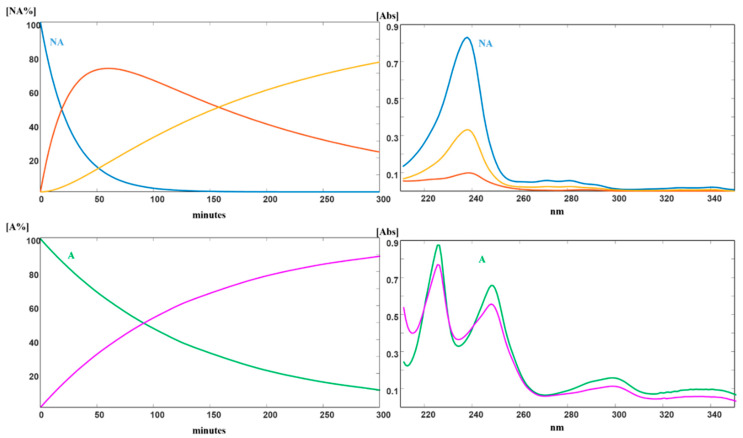
Photodegradation of NA and A: kinetic profiles and absorbance spectra of the pure compounds and respective photodegradation products.

The spectral data, as averages collected during five photodegradation experiments, were used to build the data matrix to be analyzed by MCR. All the measured relative standard deviation values fell within the range: 1.36%–4.79%. Figure 3 showed the photodegradation profiles of NA and A and the predicted absorbance spectra of their photoproducts.

The degradation process proceeded via a first-order kinetics described by the Equation:

ln[%DRUG]=−kt + 4.6(1)

where % DRUG was the percentage of residual drug, k the photodegradation rate constant, t the time (min), and 4.6 the logarithm of the starting concentration (100%). The parameters t_0.1_ (time to cause 10% degradation) and t_0.5_ (time to cause 50% degradation) were used to compare the degradation behavior of the tested formulations.

ME formulations of each drug were prepared as above described and subdued to photodegradation test. The kinetic profiles were calculated also in these experiments. Table 2 lists the kinetics parameters calculated for both compounds in solution and ME liquid formulations.

### 3.3. Photodegradation of Gel Formulations

Photodegradation tests were applied on the gel formulations prepared as above described. Gel formulations were made with 0.2 and 0.7% of pure NA and 0.2% of pure A. All the prepared systems were exposed to light.

**Figure 4:**
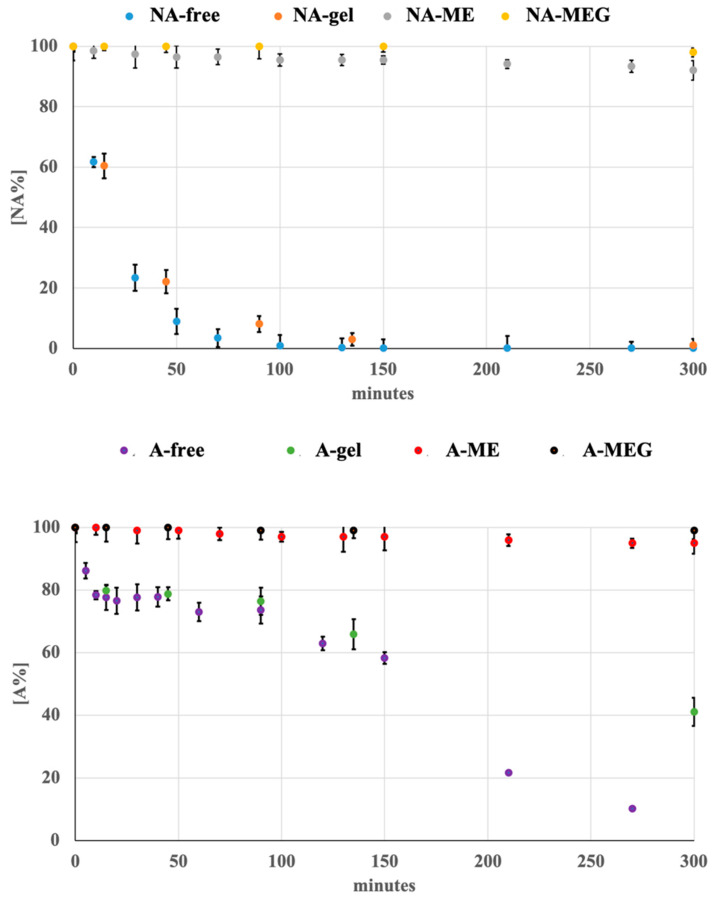
Photodegradation profiles of NA and A in all the tested formulations.

The stability of the compounds was also investigated by preparing the ME-drug in gel with 0.2% of the drug. The data obtained from the photodegradation experiments were processed by MCR and the kinetics parameters were calculated. The photodegradation profiles of these formulations followed a first-order kinetics. Table 2 summarizes the degradation rate constants and the values of t_0.1_ and t_0.5_ for the tested matrices. In Figure 4, the photodegradation profile of all the ME and MEG formulations were compared with the ethanol solutions and plain gels for NA and A, respectively.

### 3.4. Ex-Vivo Permeation Studies

In order to evaluate the influence of the different formulations on the drug skin permeation, ex-vivo percutaneous experiments were carried out using a Franz cell diffusion system.

**Figure 5:**
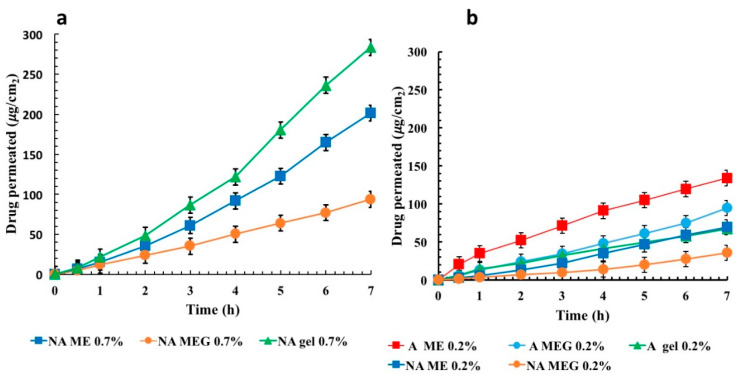
Cumulative amount of NA (**a**) and A (**b**) permeated from different samples through rabbit ear skin at 37 °C: ME (■), MEG (●), plain gel (▲) (mean ± SD; *n* = 3).

The cumulative amounts of NA and its analog A permeated across rabbit ear skin were plotted as a function of time and reported in Figure 5a,b, respectively. Both drugs were able to permeate rabbit ear skin in vitro and the cumulative amount of permeated drug increased with the time for all the samples.

**Figure 6:**
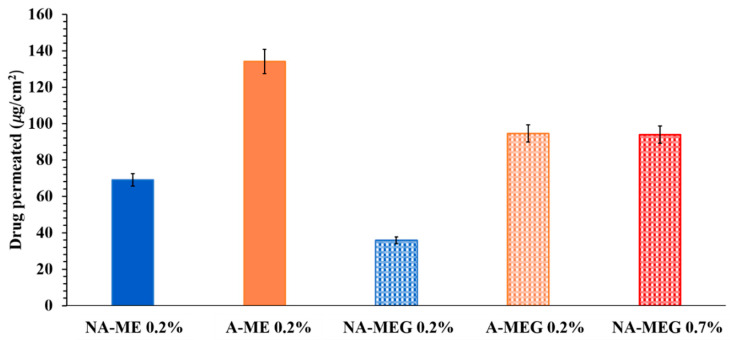
Amount of drug permeated across rabbit ear skin from different samples after 7 h at 37 °C. Values represent means ± S.D. (*n* = 3).

The skin delivery performance of the different samples was also compared and the results are summarized in Figure 6.

The calculated percentages for the applied dose after 7 h were 30.11%, 21.3%, and 15.16% for A-ME 0.2%, A-MEG 0.2%, and A gel 0.2%, respectively and 8.35%, 12.30%, and 17.33 % for NA-MEG 0.7%, NA-MEG 0.7%, and NA gel 0.7%.

## 4. Discussion

In this work, photostability of NA was investigated in liquid and gel formulations. In accordance with the results reported in literature [4,5], the obtained data confirmed the formation of 6-methoxy-2-naphthaldehyde as the major photoproduct and traces of a second by-product, as depicted in Figure 3. When the liquid formulation of NA was exposed in a light cabinet to an irradiance power of 350 W/m^2^, a full degradation of the drug was observed after about 30 min. The value of k was 0.0482 and t_0.1_ resulted to be 2.08 min, as reported in Table 2. The gel formulation of the drug containing 0.2% and 0.7% of pure NA also resulted very sensitive to light in both concentrations, showing a rapid degradation of the drug with t_0.1_ values of 4.27 and 4.37 min, respectively. Even in these cases, the formation of the same photoproducts was verified by MCR elaboration.

The photoprotection of the drug was achieved by adopting two different strategies. Firstly, a new analog (A) of NA was designed and synthesized to improve both drug stability and pharmacokinetic profile. NA is a prodrug, which is gradually metabolized to the active metabolite 6-MNA, a quite selective cyclo-oxygenase-2 inhibitor. This drug, as other NSAIDs, exerts analgesic, anti-inflammatory, anti-platelet aggregation and antipyretic activity. Due to its prolonged half-life (up to 74 h), that allows a once-daily administration, NA is one of the most used remedies to reduce pain and inflammation in patients affected by rheumatoid arthritis or osteoarthritis. After the metabolic transformation of NA, 6-MNA does not enter enterohepatic circulation. On the other hand, still active glucuronide-conjugated metabolites are excreted in urine and are present in remarkable amounts in the synovial fluid, which represents the site of action of agents used in the treatment of chronic inflammatory rheumatic diseases [43].

In view of the NA metabolic transformation and photostability profile, showing the formation of active 6-MNA and 6-methoxy-2-naphthaldehyde, respectively, the new analog A was synthesized by including part of the too light-sensitive linear side chain in an additional fused ring. This alteration should enhance light stability, while maintaining the capability to undergo metabolic activation.

**Figure 7:**
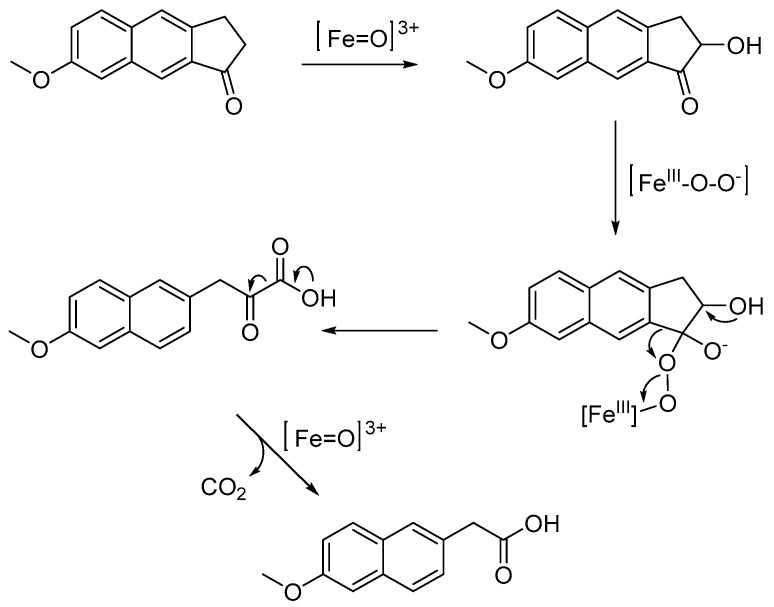
Hypothetic mechanism for the Cytochrome P450-catalyzed metabolism of A.

Accordingly, a putative mechanism for the Cytochrome P450-catalyzed activation of A is proposed in Figure 7. Similarly to what reported for NA [44,45], compound A would be subjected to oxidative transformation giving rise to the active metabolite 2-(6-methoxynaphthalen-2-yl)acetic acid after a successive double cleavage carbon-carbon bonds, by the release of carbon dioxide [46].

As expected, the liquid and gel formulation (0.2%) of A showed a far greater light stability than NA, with t_0.1_ values of 14.49 and 38.46 min (2.08 and 4.27 for NA). MCR elaboration (Figure 3), applied on the ethanol solution of A, showed the formation of one photoproduct and a degradation of 50% after about 100 min of light exposure. This photodegradation profile was confirmed also for the gel formulation with a t_0.5_ value of 265.4 min.

After these experiments, which accounted for an overall higher light stability of A, a second strategy to further stabilize the two drug molecules was designed. Accordingly, NA and A were included into suitable protective incorporation systems. The interest of pharmaceutical industry in the design of novel dosage formulations for photosensitive drugs has clearly increased over the last years. The drug incorporation in ME or MEG represents a good approach to increase drug stability and, at once, ensure optimal permeation across the skin. These systems have shown interesting and attractive advantages like high thermodynamic stability, ability to deliver both hydrophilic and lipophilic drug, increase of drug solubility and, not least, a photoprotective capability.

The tested compounds showed a clear decrease of degradation, with a very successful t_0.1_ value of about 500 min in both ME formulations. In addition, when these light-stable formulations were incorporated in gel, complete photoprotection of the drugs was observed, as depicted in Figure 4.

The percutaneous permeation profiles of the photo-stable formulations were thus investigated using Franz diffusion cells systems. As expected, skin permeation of both NA and A by MEG appeared delayed when compared to that obtained by liquid ME. Indeed, the high viscosity of the polymeric network commonly slows the drug diffusion but makes ME suitable for topical use. Different trends related to permeation through the skin were observed for NA and A. In the case of NA, skin permeation through ME systems was lower than the plain gel, which was used as control. It can be assumed that NA located in the inner phase of ME must firstly be partitioned between the oil droplets and the continuous aqueous phase and then on the skin. This led to a delayed drug permeation and higher drug retention capacity compared to plain gel, where the NA is only embedded into polymeric network and so more available. This confirmed the previous results reported in the literature: ME may control and prolong the NA release and create drug reservoir into the skin from which the drug is released slowly for a long time [42]. Such a behavior suggests that the designed formulations could be used as long-lasting delivery systems of drugs. The creation of a drug reservoir into the skin can reduce the frequency of applications, thus improving patient compliance.

Concerning A, the permeation by ME and MEG was always higher than that obtained with plain gel. Therefore, ME would act as a percutaneous permeation enhancer. This effect could be explained by the different affinity of the drug with the internal phase of ME [47].

As previously mentioned, the chemical modification reduced the hydrophobicity of A and limited its concentration in the ME formulation to a maximum 0.2%. So, the lower hydrophobicity of A influenced its solubility in the external phase and consequently the thermodynamic activity [48], which is the strength of a drug to move from formulation to skin [22]. The compound A probably diffuses more quickly from internal phase to outer phase and, consequently, shows a better partitioning inside the stratum corneum. On the other hand, NA reached a comparable permeation rate at a concentration of 0.7%, so demonstrating that the designed chemical alteration resulted favorable.

As shown in Figure 6, the permeation of A both from ME and MEG 0.2% was found, respectively, 1.94 and 2.64 higher than that obtained for NA. Moreover, a permeation rate of A 0.2% in MEG after 7 h (94.46 µg/cm^2^) was comparable with that recorded for NA 0.7% in the same system (93.87 µg/cm^2^), confirming a better performance of the new compound. This makes possible the use of lower drug doses avoiding more toxic effects.

## 5. Conclusions

The anti-inflammatory drug Nabumetone, through tests defined by international rules, has been confirmed to undergo extensive photodegradation in both solution and gel formulation. When subjected to an irradiation power of 350 W/m^2^, corresponding to 21 kJ/m^2^ min, and at a constant temperature of 25 °C, the drug resulted in degradation of 10% in only 2.08 and 4.27 min, in ethanol and gel, respectively. The properties of a new analog A, potentially endowed with higher light-stability and a more favorable pharmacokinetic profile, have been also investigated. Photoprotection approaches have been studied to realize topical light-stable formulations for both compounds, including entrapment into microemulsions. A very satisfactory time of 500 min to detect 10% degradation and an almost complete photoprotection was achieved for both compounds when their microemulsion was included in gel. The obtained results clearly suggest that the proposed microemulsions and microemulsion-in-gel as topical dosage form of Nabumetone may be potentially useful as a long-lasting drug delivery system and could be extended to the analog A for a potential transdermal use of this molecule. The overall data demonstrated that the developed formulations are effective for a controlled topical delivery of the tested compounds and that this approach could be extended for the preparation of innovative local or systemic pharmaceutical formulations.

## Figures and Tables

**Table 1:** Oil droplet size and polydispersity index (PDI) of NA and A in a microemulsions (ME) at different drug concentrations, after 1 day and 6 months after preparation, at 25 °C. Values are expressed as mean ± S.D. (*n* = 3).

Samples	Diameter Droplet (nm)	PDI	Diameter Droplet (nm)	PDI
	After 1 Day		After 6 Months	
ME	20.16 ± 1.70	0.214	20.87 ± 1.05	0.223
NA-ME 0.2%	13.17 ± 0.50	0.158	13.01 ± 0.65	0.163
NA-ME 0.7%	23.64 ± 3.21	0.169	24.52 ± 2.74	0.175
A-ME 0.2%	13.71 ± 0.32	0.153	13.54 ± 0.45	0.162

**Table 2:** Degradation kinetic parameters calculated for NA and A in different samples.

Samples		K × 10^−3^	t_0.1_ (min)	t_0.5_ (min)	R^2^
NA liquid formulations	NA-free	48.2	2.08	14.31	0.999
	NA-ME	0.20	500.95	-	0.910
NA semisolid formulations	NA-gel 0.2%	23.4	4.27	29.49	0.977
	NA-gel 0.7%	22.9	4.37	30.56	0.985
	NA-MEG	0.005	-	-	0.947
A liquid formulations	A-free	6.9	14.49	100.00	0.903
	A-ME	0.2	500.00	-	0.945
A semisolid formulations	A-gel	2.6	38.46	265.40	0.951
	A-MEG	0.07	-	-	0.924

## Supplementary Materials

The following are available online at https://www.mdpi.com/1999-4923/12/5/423/s1, Figure S1: Chemical and Analytical data of compound A, Figure S2: Copy of the 1H-NMR spectra of compound A. Figure S3: Copy of the 13C-NMR spectra of compound A.
