# Supplementary material for: Gel Formulation of Nabumetone and a Newly Synthesized Analog: Microemulsion as a Photoprotective Topical Delivery System
Source: Pharmaceutics. 2020 May 5;12(5):423. doi: 10.3390/pharmaceutics12050423 (PMC7284650; doi:10.3390/pharmaceutics12050423)
Supplement: Supplementary file 1 [file pharmaceutics-12-00423-s001.pdf]

pharmaceutics

Supplementary Materials: Gel Formulation of Nabumetone and a Newly Synthesized Analog: Microemulsion as a Photoprotective Topical Delivery System

Fedora Grande, Gaetano Ragno, Rita Muzzalupo, Maria Antonietta Occhiuzzi, Elisabetta Mazzotta, Michele De Luca, Antonio Garofalo and Giuseppina Ioele

1. Chemical and Analytical Data of Compound A

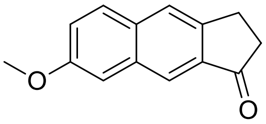

Figure S1. 7-methoxy-2,3-dihydro-1*H*-cyclopenta[*b*]naphthalene-1-one.

Mp = 152–153 °C (cyclohexane).

^{1}H-NMR (300 MHz, $CDCl_3$) δ (ppm): 2.73 (m, 2H); 3.12 (m, 2H); 3.90 (s, 3H); 7.17 (d, 1H, *J* = 1.2 Hz); 7.29 (dd, 1H, *J* =7.2, 1.2 Hz); 7.42 (d, 1H, *J* = 7.2 Hz); 7.88 (d, 1H, *J* = 7.2 Hz); 9.03 (d, 1H, *J* = 7.2 Hz).

^{13}C-NMR (75 MHz, $CDCl_3$) δ (ppm): 25.9, 36.9, 55.3, 106.7, 120.7, 124.5(×2C), 125.3, 131.0, 134.0, 134.6, 155.9, 157.9, 207.7.

IR (neat) ν_{max}:1680 cm^{-1}

GC MS (EI, 70 eV) (m/z): 213.09 ($M+H^+$).

Elemental Analysis for $C_{14}H_{12}O_2$: Calcd C, 79.22; H, 5.70; O, 15.08. Found C, 79.43; H, 5.59.

2. ^{1}**H-NMR Compound A.**

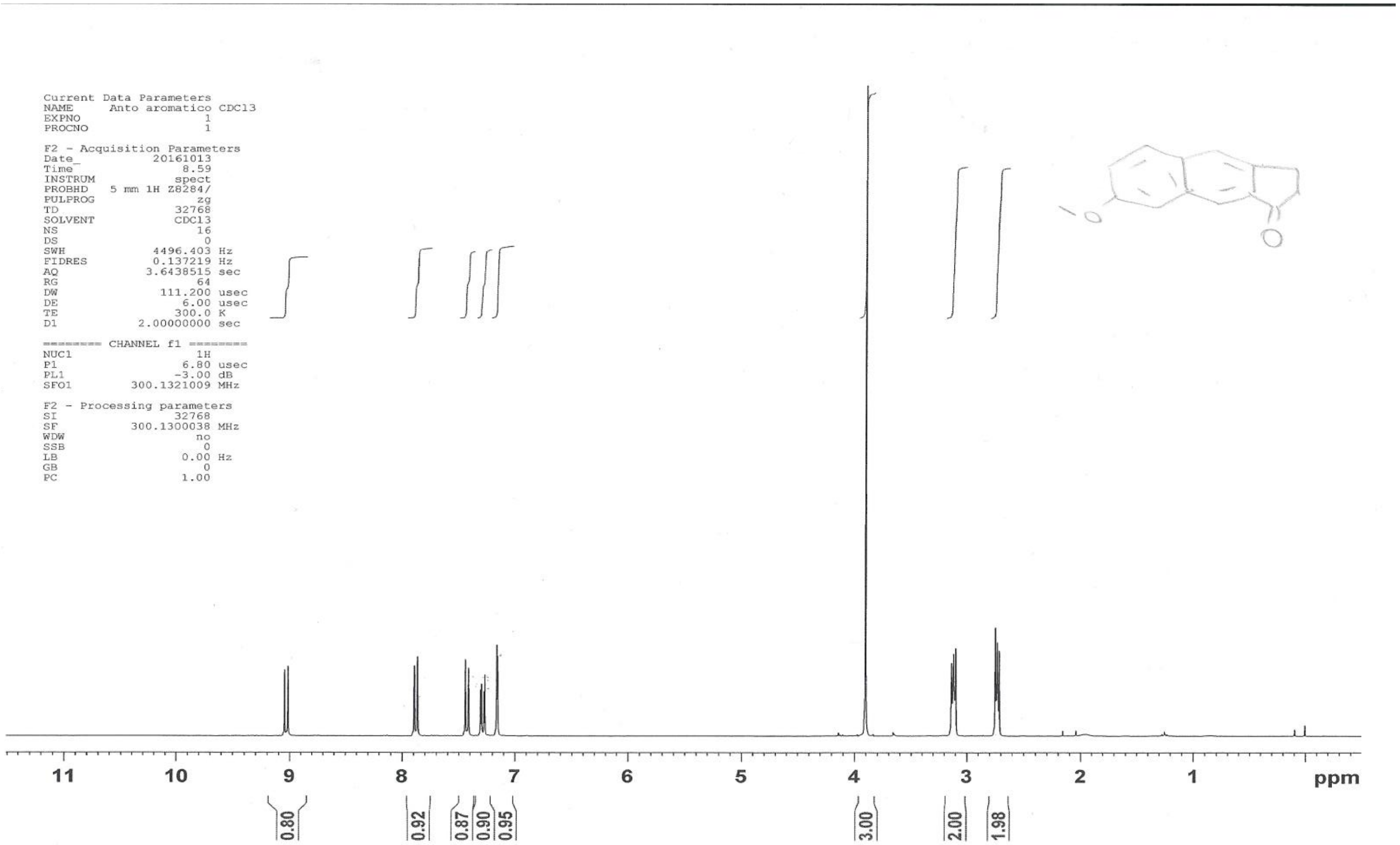

Figure S2. Copies of the ^{1}H-NMR spectra of compound A.

3.13. C-NMR Compound A

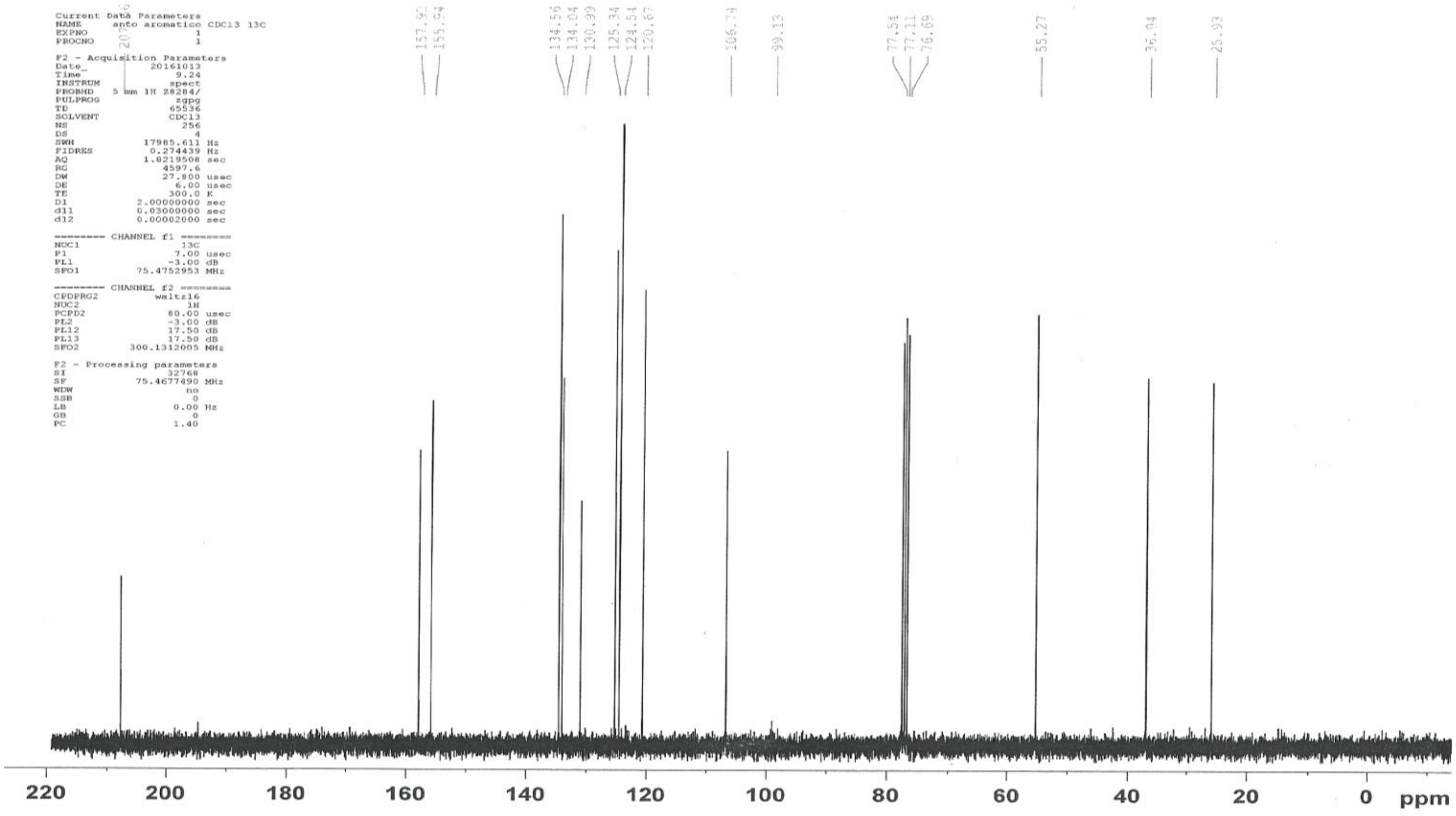

Figure S3. Copies of the ^{13}C-NMR spectra of compound A.
